# First validation of the Prostatype® P‐score in an Asian cohort: Improving risk stratification for prostate cancer

**DOI:** 10.1002/bco2.70026

**Published:** 2025-05-29

**Authors:** See‐Tong Pang, Po‐Hung Lin, Emelie Berglund, Lidi Xu, I‐Hung Shao, Kai‐Jie Yu, Chin‐Hsuan Hsieh, Tzu‐Hsuan Chang, Yu Chen, Wen‐Hui Weng, Cheng‐Keng Chuang

**Affiliations:** ^1^ Division of Urology, Department of Surgery Chang Gung Memorial Hospital, Linkou Branch Taoyuan Taiwan; ^2^ School of Medicine, College of Medicine Chang Gung University Taoyuan Taiwan; ^3^ Graduate Institute of Clinical Medical Science, College of Medicine Chang Gung University Taoyuan Taiwan; ^4^ Prostatype Genomics AB Solna Stockholms Län Sweden; ^5^ School of Medicine National Tsing Hua University Hsinchu Taiwan; ^6^ Department of Chemical Engineering and Biotechnology and Graduate Institute of Biochemical and Biomedical Engineering National Taipei University of Technology Taipei Taiwan

**Keywords:** biomarkers, mortality, prostate cancer, risk stratification, therapy

## Abstract

**Objectives:**

To evaluate the prognostic performance of the Prostatype® score (P‐score) in the Asian prostate cancer (PCa) cohort and to assess its ability to refine risk stratification compared to the National Comprehensive Cancer Network (NCCN) guidelines. This study aimed to determine whether the P‐score, previously validated in European populations, maintains its predictive accuracy in a genetically and clinically distinct high‐risk Asian cohort, where late‐stage diagnosis is more common.

**Patients and methods:**

This retrospective study included 148 PCa patients diagnosed at Taiwan Chang Gung Memorial Hospital between 2012 and 2017. Of these, 56 had primary metastases at diagnosis. The P‐score was calculated based on gene expression in core needle biopsies and clinical data collected from patients' medical records. The primary endpoint was PCa‐specific mortality (PCSM). The secondary endpoints were adverse pathology (AP) and biochemical failure.

**Results:**

The P‐score significantly outperformed NCCN in predicting PCSM, achieving a higher C‐index (0.90 vs. 0.73, P < 0.005), which reflects superior prognostic accuracy. Notably, 19.6% of patients were reclassified into different risk categories compared to NCCN, improving risk stratification and potentially altering treatment decisions for nearly one in five patients. The P‐score was also an independent predictor of adverse pathology (P = 0.003, AUC: 0.81) and biochemical failure (P = 0.03, AUC: 0.89).

**Conclusions:**

This study validated the P‐score for the first time in a non‐European population, confirming its predictive power in an Asian high‐risk setting. The reclassification of 19.6% of patients suggests that the P‐score refines risk stratification beyond NCCN, offering a more precise distinction between favourable and unfavourable outcomes, enabling more informed treatment decisions. These findings highlight the global applicability of the P‐score and its potential to improve risk assessment and personalized treatment for PCa patients worldwide.

## 1 INTRODUCTION

Prostate cancer (PCa) is the second most common cancer worldwide, accounting for 14.1% of newly diagnosed cancers in men.^1^ While advances in early detection and treatment have improved outcomes for many patients, effective management of PCa remains challenging due to the complexity of risk stratification. Inadequate stratification can lead to both over‐treatment and under‐treatment, resulting in unnecessary harm or insufficient disease control.^2^, ^3^ Notably, long‐term follow‐up studies of patients with localized PCa have demonstrated comparable prostate cancer‐specific mortality (PCSM) rates among those managed with active surveillance (AS), radical prostatectomy (RP) or radiotherapy (RT). This finding underscores the critical need for tools that can better personalize treatment strategies, balancing potential benefits with risks.^4^, ^5^ Existing risk stratification systems primarily rely on clinicopathological factors, such as prostate‐specific antigen (PSA), clinical tumour (cT) stage and Gleason pattern, to categorize patients into risk groups that guide treatment decisions. Radiographic tools, including the Prostate Imaging Reporting and Data System (PIRADS) for multiparametric MRI, are also commonly employed to refine diagnosis and stratification.^6^, ^7^ The National Comprehensive Cancer Network (NCCN) guidelines, widely used in the United States, define intermediate‐risk PCa as either favourable (suitable for AS) or unfavourable, requiring more aggressive treatment. However, significant heterogeneity in outcomes within intermediate‐risk groups highlights the limitations of current systems in accurately predicting prognosis.^8^, ^9^, ^10^, ^11^, ^12^, ^13^

The integration of genetic biomarkers into risk‐scoring systems represents a promising advancement, enhancing the predictive accuracy of traditional nomograms.^14^, ^15^ The Prostatype® score (P‐score) is one such innovative tool, designed to improve the accuracy of risk stratification and guide treatment decisions for PCa patients.^16^, ^17^ The P‐score combines a three‐gene signature (*IGFBP3*, insulin‐like growth factor binding protein 3; *F3*, coagulation factor III; *VGLL3*, vestigial‐like family member 3) with clinicopathological parameters (PSA, Gleason pattern and cT stage) to predict the risk of PCSM at diagnosis. The three‐gene signature is assessed from core needle biopsies (CNBs), and each gene has demonstrated independent prognostic value in PCa.^16^, ^18^, ^19^

The P‐score has been validated in multiple Swedish cohorts demonstrating superior accuracy in predicting PCSM and treatment outcomes.^16^, ^17^, ^20^ In Europe, PCa is typically diagnosed at early stages due to widespread PSA screening programs. Due to different screening practices, PCa diagnosis in many Asian countries, including Taiwan, often occurs at a more advanced stage^21^, ^22^ which may impact the performance of prognostic biomarkers. This is the first study to validate the P‐score in an Asian population and to assess its prognostic value in a predominantly high‐risk cohort. Given the genetic, clinical and epidemiological differences between Asian and European populations, it is critical to determine whether the P‐score can maintain its predictive accuracy across diverse patient groups. By comparing the P‐score to the NCCN risk classification system in an Asian setting, we aimed to explore its global applicability and clinical relevance for treatment decision‐making in high‐risk patients. Given that current risk stratification tools like NCCN were developed based on Western populations, this study addresses a critical gap in PCa risk assessment by testing the P‐score in a distinct genetic and clinical setting.

## 2 PATIENTS AND METHODS

### 2.1 Study cohort

**FIGURE 1:**
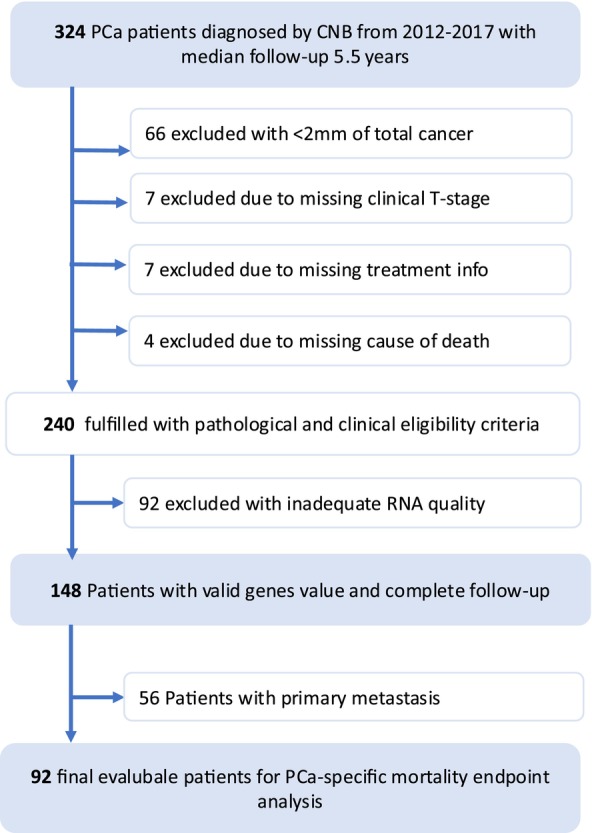
Cohort selection in the study.

This retrospective study was performed at the Taiwan Chang Gung Memorial Hospital. The cohort selection is described in supplementary material and depicted in Figure 1.

This study complied with the Declaration of Helsinki. Ethical approval was granted by the Ethics Committee in Taiwan (approval number 202200176B0C601). Patient consent to use archived biopsy material and medical records was not required by the committee due to the retrospective nature of the study. All data were anonymized and maintained with confidentiality throughout the study.

### 2.2 Specimen collection and handling

Suitable diagnostic formalin‐fixed paraffin‐embedded prostate CNB samples were identified and retrieved for use in this study according to the original pathology report. Samples were processed as previously described^16^ (see supplementary material).

### 2.3 Gene expression analysis and P‐score calculation

Expression levels of the genes IGFBP3, F3 and VGLL3 were assessed with the Prostatype® RT‐qPCR kit, and the P‐score was calculated as described in the supplemental material and in Söderdahl et al.^16^ Patients were classified into three P‐score risk groups (low‐, intermediate‐ and high‐risk) based on the calculated score.

### 2.4 Endpoints

The primary endpoint of the study was PCSM. The secondary endpoints, AP and biochemical failure were explored for patients that underwent RP. The relationship between percentage positive biopsy cores (PPB) and P‐score risk groups was analysed as an explorative outcome in patients without metastasis at diagnosis (combined dataset consisting of 96 patients from the Taiwan cohort and 316 patients from a Swedish cohort^20^).

### 2.5 Statistical analysis

The statistical analyses employed to assess the P‐score in relation to endpoints are provided in the supplemental material. A P‐value <0.05 was deemed statistically significant.

## 3 RESULTS

### 3.1 Patient characteristics

One hundred and forty‐eight patients included in the study had complete follow‐up data. Of the 92 patients without metastases at diagnosis (Table 1), 44.6% were in the NCCN risk group very high risk, 23.9% were in high‐risk, 26.1% in unfavourable intermediate‐risk and 5.4% in favourable intermediate‐risk group. In patients without metastases at diagnosis, the P‐score ranged between 2 and 14 (median P‐score 8.00). There was a wide peak between P‐score 4 and P‐score 13 with 7 to 10 patients per P‐score unit, except for P‐score 9 which was assigned to only 4 patients (Figure S1A). In patients with metastases at diagnosis (n = 56), the P‐score distribution was shifted towards higher values, ranging from 7 to 14 with a median P‐score of 12.00 (P < 0.001; Mann–Whitney U test) and a single peak at P‐score 13 (n = 19) (Figure S1B).

**TABLE 1:** Clinical characteristics of the subgroup of patients without metastases at diagnosis (n = 92).

	Controls alive at last follow‐up		PCSM		Other cause of death		Total	
N (%)	63	68.5%	8	8.7%	21	22.8%	92	100.0%
**Age at diagnosis, years**								
Median (Q1‐Q3)	71 (63–75)		78 (74–83)		76 (73–82)		73 (65–77)	
**ISUP, N (%)**								
1	10	15.9%	0	0.0%	4	19.0%	14	15.2%
2	13	20.6%	1	12.5%	1	4.8%	15	16.3%
3	15	23.8%	1	12.5%	5	23.8%	21	22.8%
4	8	12.7%	0	0.0%	6	28.6%	14	15.2%
5	17	27.0%	6	75.0%	5	23.8%	28	30.4%
**PSA at diagnosis, ng/ul**								
Median (Q1‐Q3)	10.6 (6.9–31.5)		37.4 (29.2–204.3)		22.3 (12.3–51.7)		18.3 (9.0–46.8)	
**PSA at diagnosis, N (%)**								
[0,6]	12	19.0%	0	0.0%	0	0.0%	12	13.0%
(6,10]	13	20.6%	0	0.0%	3	14.3%	16	17.4%
(10,20]	17	27.0%	0	0.0%	6	28.6%	23	25.0%
(20,30]	4	6.3%	2	25.0%	2	9.5%	8	8.7%
>30	17	27.0%	6	75.0%	10	47.6%	33	35.9%
**Clinical N stage, N (%)**								
0	57	90.5%	5	62.5%	20	95.2%	82	89.1%
1/1b	6	9.5%	3	37.5%	1	4.8%	10	10.9%
2								
na								
**Clinical stage, N (%)**								
1	0	0.0%	0	0.0%	1	4.8%	1	1.1%
2/2a	9	14.3%	0	0.0%	3	14.3%	12	13.0%
2b/2c	27	42.9%	0	0.0%	4	19.0%	31	33.7%
3/3a	6	9.5%	1	12.5%	3	14.3%	10	10.9%
3b	15	23.8%	5	62.5%	6	28.6%	26	28.3%
4	6	9.5%	2	25.0%	4	19.0%	12	13.0%
**Clinical M stage, N (%)**								
0	63	100.0%	8	100.0%	21	100.0%	92	100.0%
1/1a/1b/1c		0.0%		0.0%		0.0%		0.0%
**PSA relapse, N (%)**								
no	46	73.0%	3	37.5%	20	95.2%	69	75.0%
yes	17	27.0%	5	62.5%	1	4.8%	23	25.0%
**Positive biopsy, N (%)**								
<34%	6	9.5%	0	0.0%	1	4.8%	7	7.6%
≥34%	56	88.9%	8	100.0%	20	95.2%	84	91.3%
x	1	1.6%	0	0.0%	0	0.0%	1	1.1%
**Metastasis, N (%)**								
No	54	85.7%	5	62.5%	19	90.5%	78	84.8%
Yes	9	14.3%	3	37.5%	1	4.8%	13	14.1%
na	0	0.0%	0	0.0%	1	4.8%	1	1.1%
**NCCN, N (%)**								
Favourable Intermediate	5	7.9%	0	0.0%	0	0.0%	5	5.4%
Unfavourable Intermediate	20	31.7%	0	0.0%	4	19.0%	24	26.1%
High risk	14	22.2%	1	12.5%	7	33.3%	22	23.9%
Very high risk	24	38.1%	7	87.5%	10	47.6%	41	44.6%
**First‐line treatment types, N (%)**								
Radical Prostatectomy treatment (RP)	43	68.3%	1	12.5%	6	28.6%	50	54.3%
Radiotherapy (RT)	10	15.9%	3	37.5%	7	33.3%	20	21.7%
Androgen deprivation therapy (ADT only)	10	15.9%	4	50.0%	8	38.1%	22	23.9%

### 3.2 P‐score predicts PCSM

**FIGURE 2:**
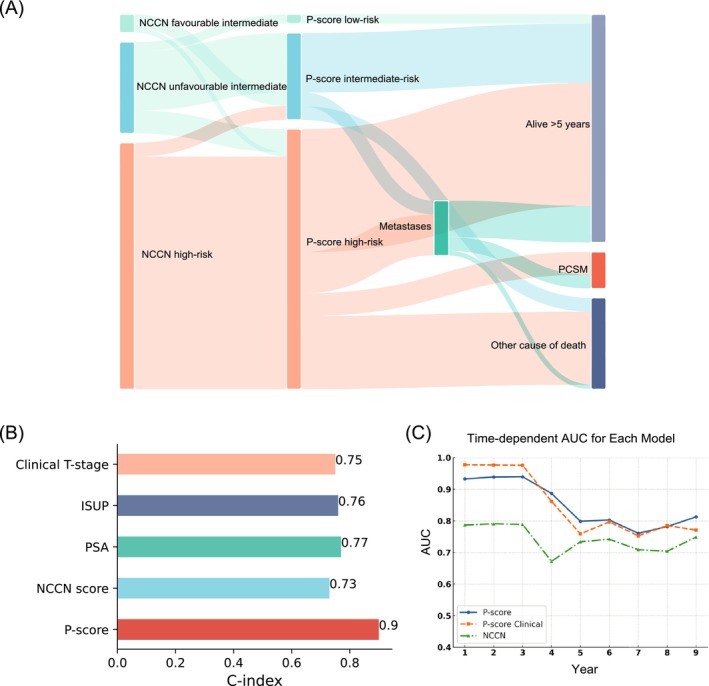
A: reclassification of NCCN favourable intermediate‐risk, unfavourable intermediate‐risk, and high‐risk prostate cancer patients with the P‐score in the subgroup of patients without metastases at diagnosis (n = 92). B: prediction accuracy of prostate cancer‐specific mortality in a C‐index analysis comparing the P‐score, NCCN score and clinicopathological parameters. C: predictive accuracy of 9‐year prostate cancer‐specific mortality for the P‐score, P‐score clinical, and the NCCN score.

Both the NCCN risk stratification system and the P‐score stratified the subgroup of patients without metastases at diagnosis (n = 92) into three different risk groups (Figure 2A and Table S1). One (20%) out of 5 patients in the NCCN risk group “favourable intermediate‐risk”, was reclassified as P‐score high‐risk. In the NCCN risk group “unfavourable intermediate‐risk”, 2 out of 24 patients (8%) were reclassified as P‐score low‐risk and 7 patients (29%) as P‐score high‐risk. Out of the 63 patients in the NCCN “high‐risk” group, the P‐score downgraded 5 patients (8%) to the P‐score intermediate‐risk group. None of the patients in the P‐score low‐ and intermediate‐risk groups had died from PCa at the last follow‐up. In the P‐score high‐risk group, 8 patients died from PCa within the follow‐up period (Figure 2A and Table S1). Three patients in the P‐score intermediate‐risk group and 10 patients in the P‐score high‐risk group developed metastases. The predictive performance of different clinicopathological parameters and the NCCN risk stratification system was compared to the P‐score in a concordance index (C‐index) analysis (Figure 2B). The C‐index of the NCCN risk stratification system was 0.73 and thus in a similar range as the individual clinicopathological parameters ISUP grade group (C‐index 0.76), cT‐stage (C‐index 0.75) and PSA value (C‐index 0.77). The C‐index of the P‐score was 0.90.

Similarly, the P‐score also outperformed the NCCN risk stratification system in predicting PCSM in a time‐dependent AUC analysis (Figure 2C). Furthermore, in order to assess the independent impact of the clinical and genetic factors that make up the P‐score, this analysis included a model, P‐score Clinical, consisting of only the clinical parameters (Gleason score, P‐stage, PSA) included in the full P‐score. The P‐score Clinical model exhibited the highest AUC at early timepoints (1–3 years), while the full P‐score model became superior at 9 years (AUC 0.771 and AUC 0.813, respectively). Randomization analysis, where either genetic or clinical variables were shuffled to determine their effect on predictive accuracy, confirmed that clinical factors are the dominant drivers of risk prediction at early timepoints, while genetic factors make a greater contribution at later timepoints. Both P‐score models consistently outperformed the NCCN system model, which exhibited the lowest predictive performance at all timepoints and was inferior to both P‐score models at 9 years (AUC 0.749) (Figure 2C).

**FIGURE 3:**
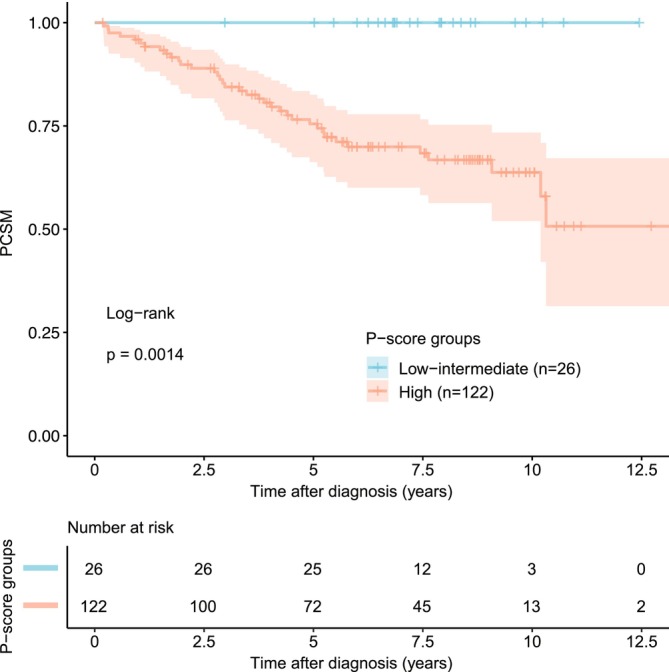
Prostate cancer‐specific survival by P‐score risk group (low‐ and intermediate‐ risk vs. high‐risk) (n = 148).

Survival analysis in the total cohort of patients showed significantly lower PCSM for patients in the low‐ and intermediate P‐score group compared to patients in the P‐score high‐risk group (P = 0.001, log‐rank test, n = 148; Figure 3). Corresponding data for the subgroup of patients without metastases at diagnosis is provided in the supplemental material (Figure S2).

### 3.3 P‐score association with adverse pathology

**FIGURE 4:**
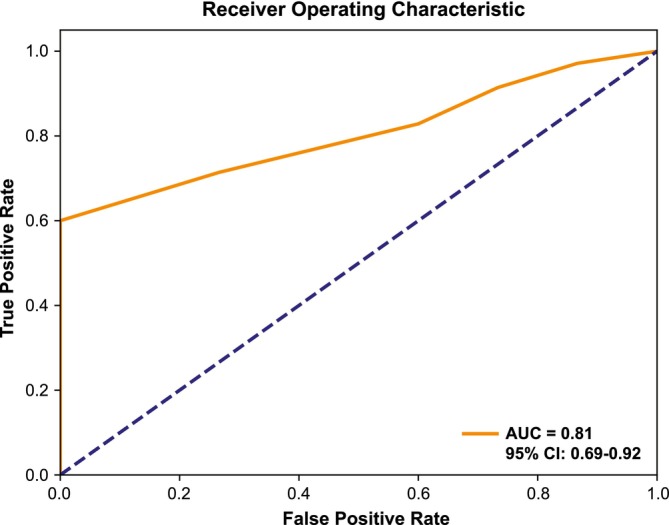
P‐score association with adverse pathology (AP) in the subgroup of patients who underwent radical prostatectomy (RP). Area under the curve (AUC) assessed by receiver operating characteristic (ROC) analysis.

The ability of the CNB‐based P‐score to predict AP (defined as pathological T‐stage [pTstage] ≥ 3a or ISUP grading ≥3 in surgical specimens) was assessed in patients who underwent RP (n = 50). In a logistic regression model, the CNB‐based P‐score predicted AP in patients that underwent RP with an odds ratio of 1.80 (95% CI: 1.21–2.67; P = 0.003) for each unit of increase in P‐score (Table 2). In ROC analysis, AUC of 0.81 (95% CI: 0.69–0.92) indicates that the P‐score can reliably identify patients with AP (Figure 4). The association between MRI‐based and pathological staging was analysed in 50 patients who underwent RP. The MRI staging was the same for 31 patients, lower for 15 patients and higher for 4 patients than pTstage. In ROC analysis, AUC for the P‐score was 0.75 (95% CI: 0.60–0.88) and for MRI T‐staging was 0.67 (95% CI: 0.56–0.79) (Figure S3).

**TABLE 2:** Prediction of adverse pathology in patients that underwent radical prostatectomy using CNB‐based P‐score (n = 50).

Parameter	P‐score
**Adverse pathology (pISUP≥3 or pTstage≥3)**	
**p‐value**	0.003
**Odds ratio (95% CI)**	1.8 (1.21–2.67)

### 3.4 P‐score predicts ***biochemical failure***

Biochemical failure refers to a rise in blood PSA levels (>0.2 ng/ml) following RP and was assessed using ROC analysis in 49 patients with eligible data. The AUC for P‐score (0.89, 95% CI: 0.76–1) was higher than for NCCN (0.75, 95% CI: 0.59–0.91), P = 0.03 (Figure S4).

### 3.5 P‐score and first‐line treatment decisions

In the cohort of patients without metastases at diagnosis (n = 92), RP was the most common first‐line treatment (54.3%), followed by androgen deprivation therapy (ADT) (23.9%) and RT (21.7%). Patients who died from PCa were most frequently treated with ADT as their initial therapy (50.0%). Notably, none of the patients in this cohort received conservative management such as AS or watchful waiting. Among the treatment groups, patients treated with RP had the highest proportion of survivors at the last follow‐up (68.3%) (Table 1).

**FIGURE 5:**
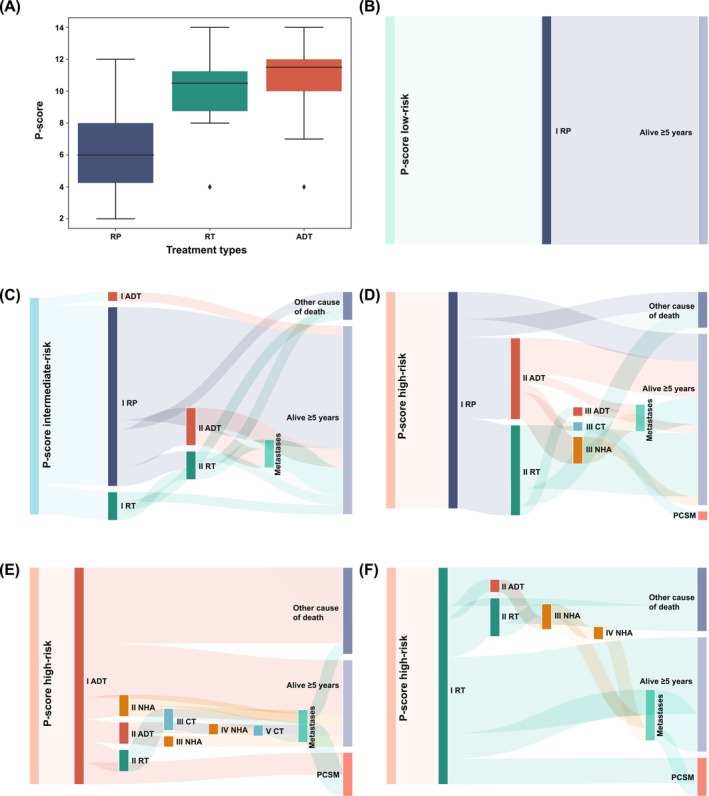
A: distribution of the P‐score across treatment groups. B: treatment and survival status of P‐score low‐risk patients. C: treatment and survival status of P‐score intermediate‐risk patients. D‐F: treatment and survival status of P‐score high‐risk patients.

To explore the relationship between P‐scores and treatment decisions, we analysed the distribution of P‐scores across first‐line treatments. Patients treated with RP had the lowest P‐scores (median [IQR]: 6^4^, ^5^, ^6^, ^7^, ^8^), while those treated with RT (median [IQR]: 10.5^9^, ^10^, ^11^) and ADT (median [IQR]: 11.5^10^, ^11^, ^12^) had higher scores (Figure 5A). This suggests that patients with higher P‐scores were more likely to receive non‐surgical therapies as initial treatment.

We further examined treatment patterns, metastasis development and survival outcomes based on P‐score risk groups in 87 patients with at least 5 years of follow‐up. Patients were classified as low‐risk (n = 3), intermediate‐risk (n = 23) or high‐risk (n = 61) based on their P‐scores. In the low‐risk group, all patients were treated with RP and were alive at the 5‐year follow‐up (Figure 5B). Among intermediate‐risk patients, RP was the most common first‐line treatment (83%), followed by RT (13%) and ADT (4%). After 5 years, 87% of intermediate‐risk patients were still alive, while 13% had died from causes unrelated to PCa (Figure 5C).

In the high‐risk group, patients received RP (39%, n = 24), ADT (33%, n = 20) or RT (28%, n = 17) as their initial treatment (Figures 5D–F). Among those who underwent RP, 38% later received ADT and 42% received RT as second‐line treatments. Metastases occurred in 13% of these patients. Five years after diagnosis, 79% were alive, 17% had died from non‐PCa causes and 4% had died from PCa (Figure 5D). Patients initially treated with ADT had poorer outcomes. Ten percent of these patients later received novel hormone agents, and another 10% underwent RT as second‐line therapy. Metastases developed in 15% of patients in this group. By 5 years, only 40% were alive, while 40% and 20% had died from non‐PCa and PCa‐related causes, respectively (Figure 5E). Among patients initially treated with RT, 6% received ADT and 18% underwent additional RT as second‐line treatments. Metastases occurred in 24% of RT‐treated patients. At 5 years, 53% of these patients were still alive, 18% had died from PCa and 29% from non‐PCa causes (Figure 5F).

This analysis highlights the association between P‐scores, treatment decisions and patient outcomes. Patients with lower P‐scores were more likely to undergo RP and had better survival outcomes, while those with higher P‐scores often received ADT or RT and experienced worse outcomes. These findings underscore the potential value of the P‐score in guiding treatment decisions and improving risk stratification.

### 3.6 Relationship between PCa tumour volume and P‐score

The PPB provides information about the extent and aggressiveness of PCa. We therefore evaluated the relationship between the PPB and the P‐score risk groups in the subset of patients without metastases at diagnosis. The analysis was based on a combined dataset (n = 412) consisting of 96 patients without metastases at diagnosis from the Taiwan cohort and 316 metastasis‐free patients from a Swedish cohort described in a previous publication^20^ (Figure S5).

The combined dataset demonstrated a strong correlation between PPB and P‐score risk groups (Spearman correlation coefficient 0.51), with a statistically significant difference in PPB distribution across P‐score risk groups (P < 0.001; Kruskal‐Wallis test). This was confirmed in a post‐hoc pairwise comparison using Dunn's test after applying the Bonferroni correction for multiple comparisons (P < 0.05). Effect size estimation for the combined dataset indicated that risk group stratification accounts for 18.7% of the variance in PPB, suggesting a strong influence.

## 4 DISCUSSION

Prior studies validating the P‐score have been conducted exclusively in European populations, where patients are often diagnosed at earlier stages due to routine PSA screening.^16^, ^17^, ^20^ This study is the first to validate the P‐score in an Asian PCa cohort, demonstrating that its predictive accuracy extends beyond European populations. Our findings confirm that the P‐score outperforms NCCN in a late‐stage, high‐risk setting, highlighting its potential as a globally applicable risk stratification tool. The reclassification of nearly 20% of patients into more appropriate risk categories suggests that incorporating the P‐score into clinical practice could optimize treatment selection and improve patient outcomes – particularly in non‐Western populations where late‐stage diagnosis is prevalent.^21^, ^22^ Our findings confirm that the P‐score retains its predictive power in this distinct clinical setting, even among patients classified as high‐risk or very high‐risk by NCCN guidelines.

We observed that survival rates decreased with increasing P‐score risk group, supporting the prognostic value of the P‐score in predicting patient outcomes. In addition, the P‐score accurately predicted PCSM and outperformed the NCCN risk stratification system in both a competing risk C‐index analysis and a time‐dependent AUC analysis. These findings are in line with earlier observations in a Swedish cohort.^16^ In the current study, the C‐index was higher than in Swedish cohorts. This can be partly explained by the large proportion of high‐risk patients included in this study, as risk stratification is generally more accurate in these patients compared to low‐ and intermediate‐risk patients. The cohort in this study is, however, representative of the Asian PCa population.

In order to assess the independent impact of the clinical and genetic factors that make up the P‐score, our time‐dependent AUC analysis over 9 years compared a full P‐score model, a NCCN classification model and the P‐score Clinical model that incorporated only the clinical parameters that form part of the full P‐score (Gleason score, P‐stage, PSA). In this analysis, both P‐score models consistently outperformed the NCCN classification, reinforcing the advantage of a structured, integrated risk assessment approach for PCa over conventional clinical staging. Comparison of the full P‐score model and the P‐score Clinical model shows that P‐score Clinical performed best at early time points, however, the full P‐score model became the superior model by 9 years. This suggests that while clinical factors drive early prognosis, the integration of genetic markers enhances predictive accuracy over longer follow‐up periods.

In this study, the P‐score reclassified 18 of the 92 patients without metastases at diagnosis (19.6%) into a different risk group, compared to the widely used NCCN risk stratification system, used in the study centre. Of these, 7 patients were downgraded to a lower‐risk group and 11 patients were upgraded to a higher‐risk group. Reclassification was most frequent for NCCN favourable intermediate‐risk patients, for which the NCCN guidelines recommend AS, although some patients may be at risk for a worse outcome.^23^ Thus, the P‐score appears to provide a more nuanced risk assessment than the NCCN categories. However, further studies in larger patient cohorts and with follow‐up beyond 10 years are required to assess whether the more refined risk stratification achieved by the P‐score significantly improves survival outcomes, particularly in light of the typically slow progression of PCa.^24^, ^25^ Although the 5‐year follow‐up in this study was rather short, the study cohort contained a large proportion of patients diagnosed at advanced stages where a faster progression could be expected and therefore is still valuable.

Patients in the P‐score high‐risk group had diverse treatment paths and outcomes which could be attributed not only to the complexity and aggressiveness of their tumours but also to the choice of therapy. Prostatectomy appears to be very effective in low‐ and intermediate‐risk groups, while high‐risk patients present more complex scenario that requires a multifaceted treatment approach.

Today, multiparametric MRI and PIRADS systems guide clinical staging and treatment planning.^26^, ^27^, ^28^ In this study, a less accurate MRI‐image‐based method was employed and some patients with MRI cT3, but actual pT2 tumours, could have been referred to RT instead of RP. P‐score outperformed MRI for predicting the reclassification of pTstage. The ability of the P‐score to predict AP was also confirmed in two analyses: ROC/AUC and logistic regression. We speculate that the ability of the P‐score to predict adverse outcomes may lead to better monitoring and potentially improved clinical outcomes for PCa patients.

Finally, in this study, we observed a strong correlation between PPB and P‐score. PPB is an inclusion parameter in some AS on the basis of early landmark studies which posited that tumour volume contributes to the aggressiveness of PCa, and showed that biopsy tumour volume is associated with clinical outcome.^29^, ^30^, ^31^, ^32^

## 5 CONCLUSIONS

This study confirms that the P‐score is a superior risk stratification tool compared to NCCN classification, and demonstrates the long‐term prognostic value provided by integrating clinical and genetic factors when performing PCa risk assessment. P‐score consistently outperformed NCCN in predicting PCSM, AP and biochemical failure, underscoring its potential as a valuable tool for personalized treatment decision‐making in Asian PCa patients. This study also highlights the broader need for prognostic biomarkers that can be applied across genetically and clinically diverse populations, addressing a key challenge in PCa management. Future research should focus on integrating P‐score‐based risk stratification into clinical practice to assess its potential for optimizing treatment selection and improving long‐term outcomes, particularly in high‐risk patient cohorts.

## AUTHOR CONTRIBUTIONS

Conceptualization and Methodology: ST.P. and E.B.; Software, Validation and Formal Analysis: L.X. and PH.L.; Investigation: E.B., L.X., Y.C. and WH.W.; Data Curation: PH. L, E.B. and L.X.; Writing – Original Draft Preparation: PH.L. and E.B.; Writing – Review & Editing: ST.P. and PH.L.; Visualization: E.B. and L.X.; Supervision: ST.P. and CK.C; Resources: IH.S., KJ.Y., CH.H., TH.C. and Y.C.; Project Administration and Funding Acquisition: IH.S., KJ.Y., CH.H. and TH.C.

## CONFLICT OF INTEREST STATEMENT

EB is employed by Prostatype Genomics AB. LDX was employed by Prostatype Genomics AB. The authors report no other conflicts of interest in this work.

## ETHICS APPROVAL

Ethical approval was granted by the Ethics Committee in Taiwan (approval number 202200176B0C601).

## PATIENT CONSENT

Patient consent to use archived biopsy material and medical records was not required by the committee due to the retrospective nature of the study.

## Supporting information


**Figure S1:** P‐score distribution. (a) Patients without metastases at diagnosis (n = 92). (b) Patients with metastases at diagnosis (n = 56).

**Figure S2:** Prostate cancer‐specific survival by P‐score risk group (low‐ and intermediate‐ risk vs. high‐risk) in patients without metastases at diagnosis (n = 92).

**Figure S3:** Prediction of pathological T‐score assessed in patients with available data who underwent prostatectomy (n = 50) by P‐score and by MRI‐based evaluation. Receiver operating characteristic (ROC) analysis showed that the area under the curve (AUC) for P‐score was 0.75 and AUC for MRI‐based T‐staging was 0.67.

**Figure S4:** Predicted biochemical failure in a subgroup of patients who underwent radical prostatectomy (RT) as first‐ or second‐line treatment. Area under the curve (AUC) for P‐score and NCCN‐score was assessed using receiver operating characteristic (ROC) analysis and the difference between AUC for the two scores was significant at 5 years follow‐up (P = 0.03).

**Figure S5:** Relationship between positive biopsy cores (PPBs) (%) and P‐score risk stratification (P‐score groups 0, 1 and 2) in a combined dataset (n = 412) consisting of 96 patients without metastases at diagnosis from the Taiwan cohort and 316 metastasis‐free patients from a Swedish cohort (Saemundsson et al 2023).

**Table S1:** Reclassification of patients in different NCCN risk groups using the P‐score in the subgroup of patients without metastases at diagnosis (n = 92).

## Data Availability

The data that support the findings of this study are available from the corresponding author upon reasonable request.
