# Supplementary figures and images for: First validation of the Prostatype® P‐score in an Asian cohort: Improving risk stratification for prostate cancer
Source: BJUI Compass. 2025 May 29;6(6):e70026. doi: 10.1002/bco2.70026 (PMC12123050; doi:10.1002/bco2.70026)

**a****Patients without metastases**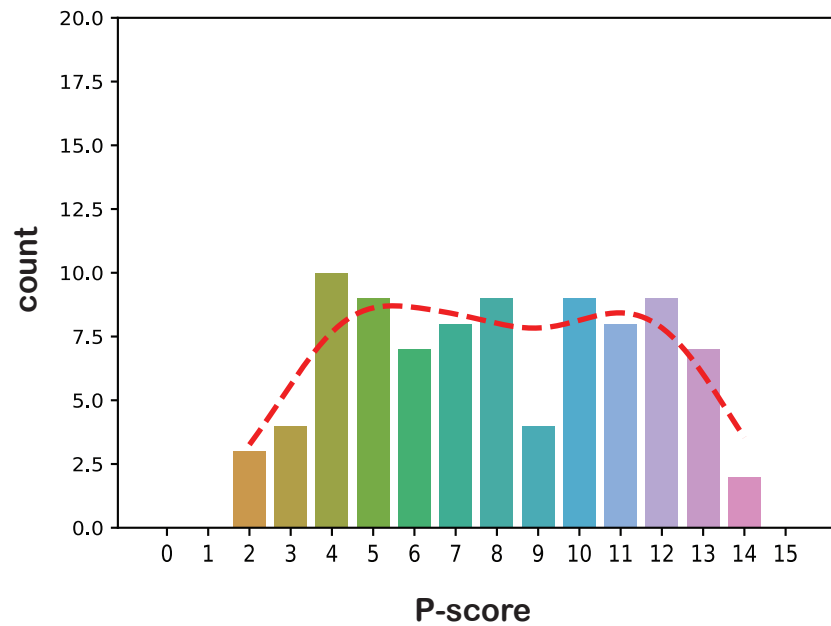**b****Patients with metastases**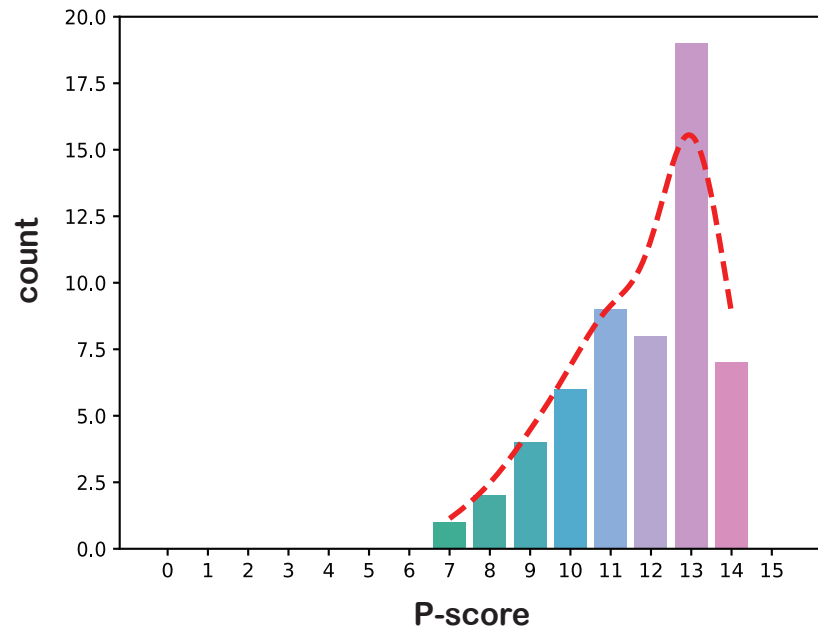

Supplement: Supplementary file 1 — Figure S1: P‐score distribution. (a) Patients without metastases at diagnosis (n = 92). (b) Patients with metastases at diagnosis (n = 56). [file BCO2-6-e70026-s005.pdf]

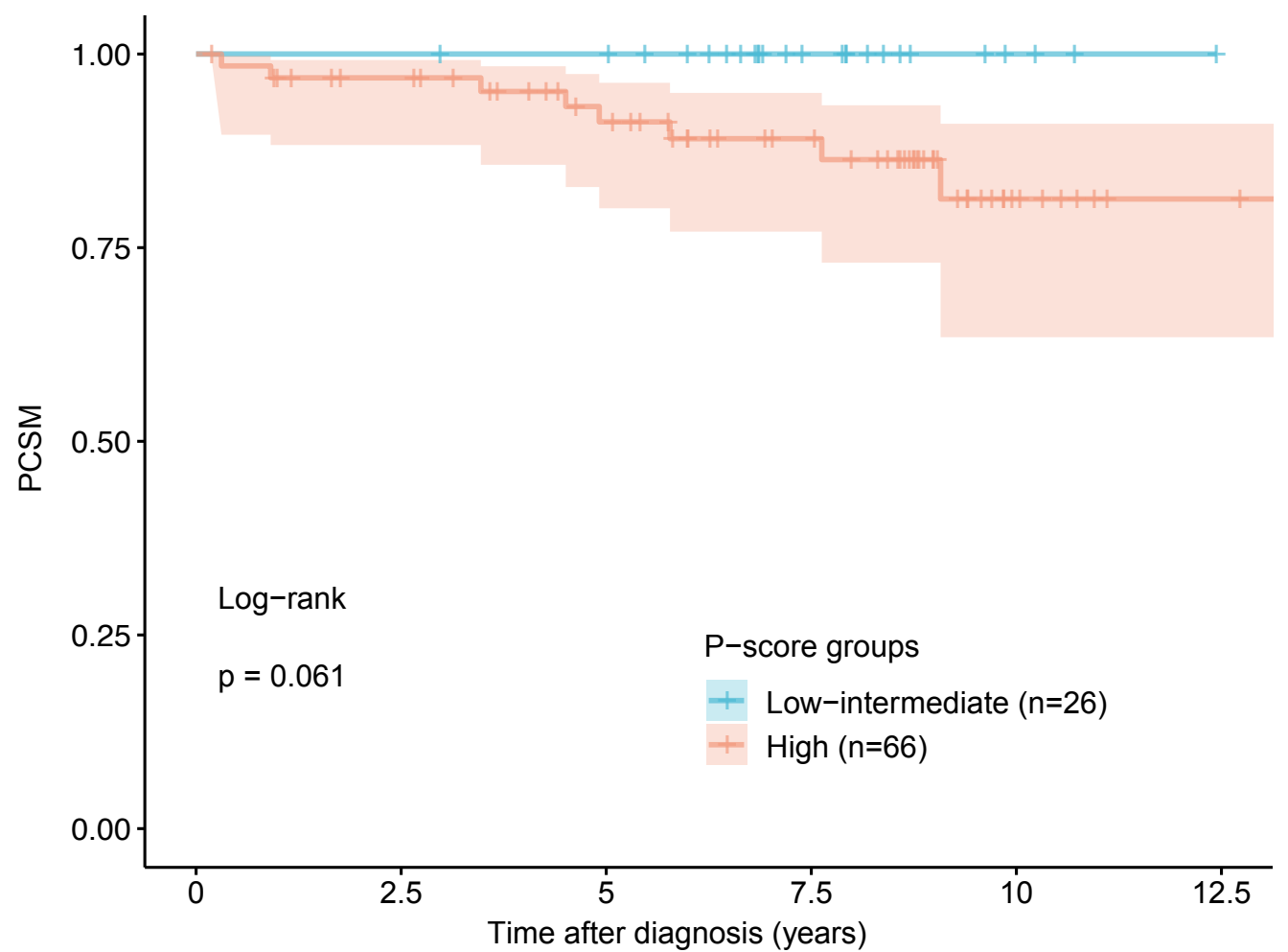

Number at risk

| P-score groups          | 0  | 2.5 | 5  | 7.5 | 10 | 12.5 |
|-------------------------|----|-----|----|-----|----|------|
| Low-intermediate (n=26) | 26 | 26  | 25 | 12  | 3  | 0    |
| High (n=66)             | 66 | 58  | 46 | 34  | 8  | 2    |

Time after diagnosis (years)

Supplement: Supplementary file 2 — Figure S2: Prostate cancer‐specific survival by P‐score risk group (low‐ and intermediate‐ risk vs. high‐risk) in patients without metastases at diagnosis (n = 92). [file BCO2-6-e70026-s003.pdf]

ROC Curves

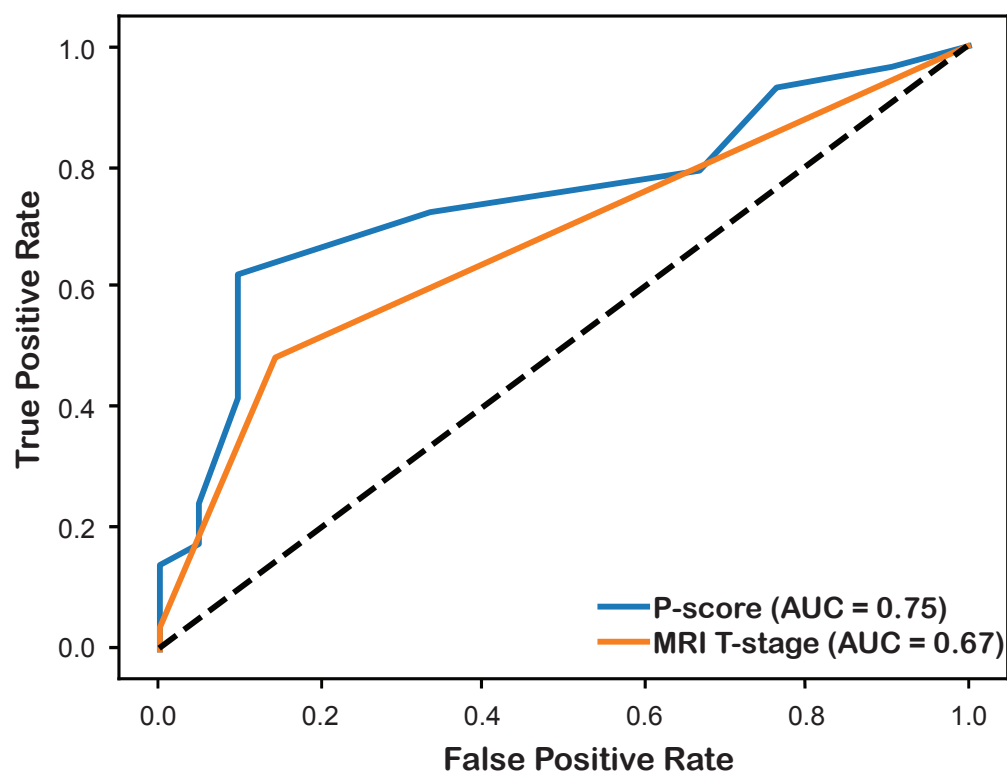

Supplement: Supplementary file 3 — Figure S3: Prediction of pathological T‐score assessed in patients with available data who underwent prostatectomy (n = 50) by P‐score and by MRI‐based evaluation. Receiver operating characteristic (ROC) analysis showed that the area under the curve (AUC) for P‐score was 0.75 and AUC for MRI‐based T‐staging was 0.67. [file BCO2-6-e70026-s001.pdf]

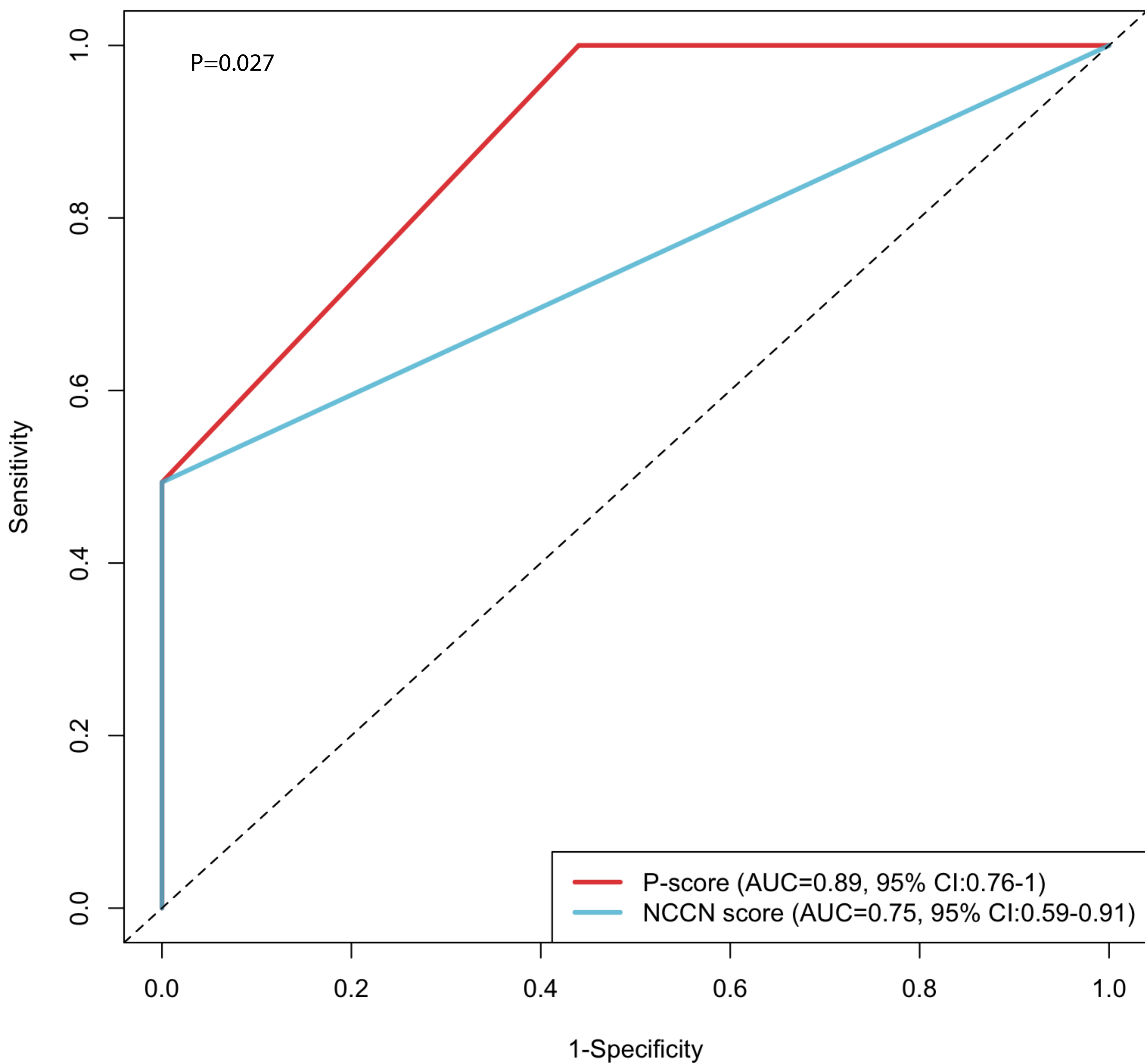

Supplement: Supplementary file 4 — Figure S4: Predicted biochemical failure in a subgroup of patients who underwent radical prostatectomy (RT) as first‐ or second‐line treatment. Area under the curve (AUC) for P‐score and NCCN‐score was assessed using receiver operating characteristic (ROC) analysis and the difference between AUC for the two scores was significant at 5 years follow‐up (P = 0.03). [file BCO2-6-e70026-s002.pdf]

Positive biopsy cores (PPBs) vs P-score groups

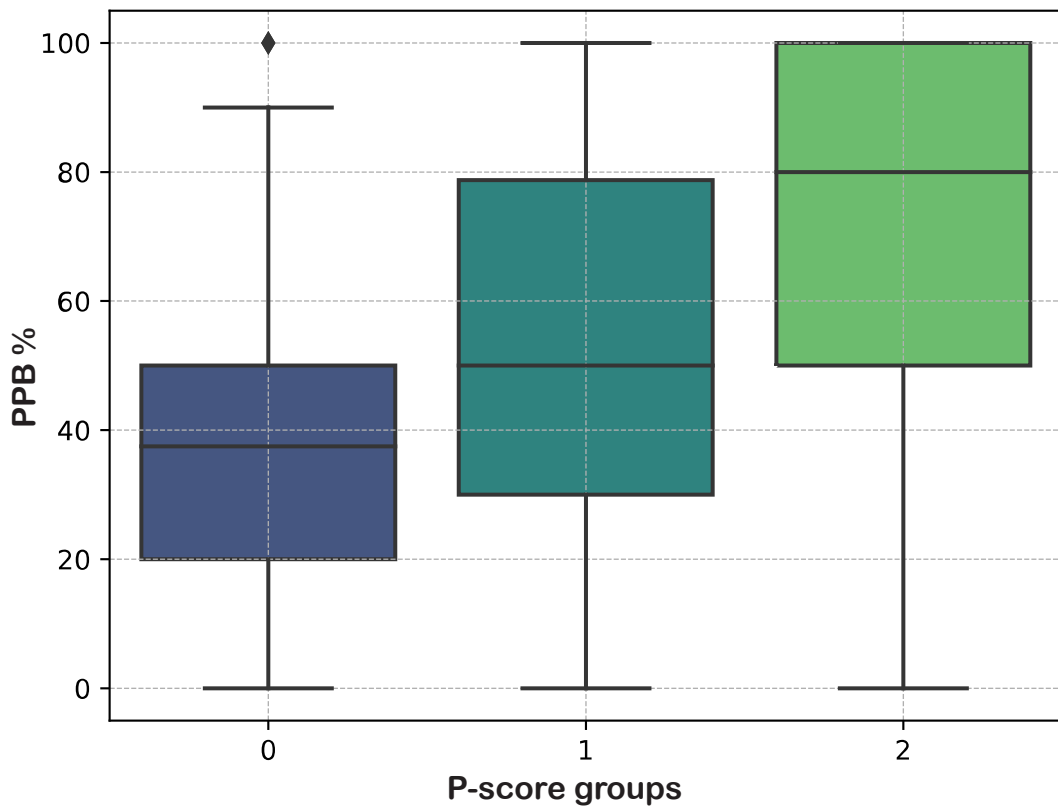

Supplement: Supplementary file 5 — Figure S5: Relationship between positive biopsy cores (PPBs) (%) and P‐score risk stratification (P‐score groups 0, 1 and 2) in a combined dataset (n = 412) consisting of 96 patients without metastases at diagnosis from the Taiwan cohort and 316 metastasis‐free patients from a Swedish cohort (Saemundsson et al 2023). [file BCO2-6-e70026-s004.pdf]
