# Supplementary material for: First validation of the Prostatype® P‐score in an Asian cohort: Improving risk stratification for prostate cancer
Source: BJUI Compass. 2025 May 29;6(6):e70026. doi: 10.1002/bco2.70026 (PMC12123050; doi:10.1002/bco2.70026)
Supplement: Supplementary file 6 — Table S1: Reclassification of patients in different NCCN risk groups using the P‐score in the subgroup of patients without metastases at diagnosis (n = 92). [file BCO2-6-e70026-s006.docx]

**SUPPLEMENTARY MATERIAL**

# Patients and methods

## Study cohort:

In this study, patient consent to use archived biopsy material and medical records was not required by the committee due to the retrospective nature of the study. All data were anonymized and maintained with confidentiality throughout the study.

Three hundred twenty four PCa patients diagnosed at the Taiwan Chang Gung Memorial Hospital between 2012-2017 were screened. In total, 240 patients fulfilled the pathological and clinical eligibility criteria (see supplemental material), and their biopsies were subjected to gene expression analysis with the Prostatype® RT-qPCR kit. Ninety-two patients were excluded due to inadequate RNA quality; 148 (62%) patients had valid test results and complete follow-up information, and were included in the analyses. Of these patients, 56 had primary metastases at diagnosis and were excluded from certain analyses, including PCSM. For the remaining patients, the median follow-up time was 6.25 years (interquartile range [IQR]: 3.72-8.69).

## Eligibility criteria cohort selection:

To be eligible for inclusion in this study, a histologically confirmed prostate cancer (PCa) diagnosis (ICD 10 code C61) was required. The following exclusion criteria applied: (i) medical records not available, (ii) Formalin-fixed paraffin-embedded (FFPE) core needle biopsy (CNB) material not available, (iii) treatment regimen unknown, (iv) age at diagnosis <50 years or >100 years, (v) total tumour length <2 mm according to the pathology report, (vi) patient died from accident, homicide or suicide, (vii) less than 50% cancer cell coverage on the area marked for gene expression analysis, and (viii) invalid gene expression analysis. Patients that fulfilled any of these exclusion criteria were not eligible for inclusion in the study.

## Formalin-fixed paraffin-embedded sample preparation and ribonucleic acid extraction from core needle biopsy samples:

FFPE prostate CNB samples were collected at the time of PCa diagnosis according to the routine procedure used at Taiwan Chang Gung Memorial Hospital. All biopsies were stored in the Linkou Chang Gung Memorial Hospital (CGMH) biobank facility under suitable conditions before use in this study. According to the original pathology report, PCa-containing specimens were identified and retrieved. For sample preparation, FFPE CNB tissue was sectioned, marked, and quantified as described previously [1]. Marked cancer-cell containing areas were scraped from unstained FFPE sections into DNase/RNase-free microcentrifuge tubes for RNA extraction using disposable scalpels for each patient’s sample. If required, tissue samples from different biopsies of the same patient were combined. The scraped cancer area was at least 15 mm^2^ with ≥50% epithelial cancer cells for most patients. Total RNA was isolated from the scraped tissue using the commercially available Maxwell® 16 LEV RNA FFPE Purification Kit (Promega, catalogue number: AS1260) according to the manufacturer’s instructions. Isolated RNA was subjected to a four-plex one-step real-time quantitative polymerase chain reaction (RT-qPCR) analysis immediately without storing.

## One-step RT-qPCR reaction and gene expression:

Total RNA was used for gene expression analysis in a four-plex one-step RT-qPCR. The expression levels of the three biomarker genes (*F3*, *IGFBP3* and *VGLL3*) and the control gene (glyceraldehyde 3-phosphate dehydrogenase, *GAPDH*) were measured using the commercially available Prostatype® RT-qPCR kit (Prostatype Genomics AB, Solna, Sweden). All measurements were conducted using a Roche LightCycler 480 instrument II (Roche Molecular Systems, Inc.), a qPCR platform on which a colour compensation method was run prior to performing the qPCR analysis. The sequence information of the respective probes and primers has been published previously [2]. The Prostatype® RT-qPCR kit contains a positive and negative control. These were analysed with each batch of RNA isolated from FFPE tissue samples and a batch was considered valid only if positive and negative controls were valid. Samples with *GAPDH* C(t) values of >28.0 were excluded from further analysis as per the manufacturer’s instructions [3]. Expression levels of the three biomarker genes (*F3*, *IGFBP3* and *VGLL3*) in each patient sample were normalised to the expression level of *GAPDH* and presented as delta C(t) (ΔC[t]) values. All samples were analysed in triplicate and the median ΔC(t) value of the triplicates for each gene used for subsequent calculations. At least two valid triplicates were required and in case one triplicate was invalid, the mean of the two remaining ΔC(t) values was used.

## Gene expression analysis and P-score calculation:

Expression levels of the genes *F3*, *IGFBP3*, and *VGLL3* were assessed with the Prostatype® RT-qPCR kit, and the P-score was calculated. The P-score is based on an algorithm which was developed using a Fine-Gray competing risk model in a retrospective cohort of historical PCa patients to calculate an individual risk score, and ranges between the integers 0-15 [4]. The P-score has been validated in three independent Swedish cohorts; a total of 663 PCa patients [4–6]. Based on predefined P-score cut-off values, patients were categorised into three P-score risk groups (low-, intermediate-, and high-risk).

## Statistical analysis:

P‐score risk groups in predicting PCa‐free survival were assessed using the Kaplan–Meier method. Univariate Cox proportional hazards models were used to calculate the concordance-index (C-index) for P-score and other clinical parameters in predicating PCSM. The prognostic performance of the P‐score in comparison with both NCCN and a model consisting of only the clinical parameters included in the P-score (P-score Clinical) was further investigated using randomization-based analysis of data from the Taiwan cohort (n=92). This approach preserved the original data distribution while assessing the independent impact of clinical and genetic factors over a 9 year follow-up period. Two randomization experiments were performed where either genetic or clinical factors were shuffled to determine their effect on predictive accuracy. Model performance was assessed using time-dependent area under the curve (AUC) values across multiple timepoints.

Ordinal logistic regression analysis was used to investigate the ability of the CNB‐based P‐score to predict adverse pathology (AP).

We employed ordinal logistic regression analysis to examine the predictive capability of the P-score in determining adverse pathological outcomes, with a focus on comparing odds ratios. To assess the accuracy of the P-score in predicting AP, we utilised receiver operating characteristic (ROC) curves.

All statistical analyses were conducted under a significance threshold, with p-values less than 0.05 deemed statistically significant. The ordinal logistic regression analyses were conducted using Python 3 in a Jupyter Notebook environment. Additionally, to visualise complex relationships and flow data effectively, we generated Sankey diagrams using the D3 JavaScript package. This approach allowed us to present multidimensional data in an intuitive format, enhancing the interpretability of our findings.

All other statistical analyses were performed using the R statistical packages (the R Foundation, version 3.6.2, Vienna, Austria).

# Supplementary figure legends

Figure S1: P-score distribution

1. Patients without metastases at diagnosis (n=92)
2. Patients with metastases at diagnosis (n=56)

Figure S2: Prostate cancer-specific survival by P-score risk group (low- and intermediate- risk vs. high-risk) in patients without metastases at diagnosis (n=92)

Figure S3: Prediction of pathological T-score assessed in patients with available data who underwent prostatectomy (n=50) by P-score and by MRI-based evaluation. Receiver operating characteristic (ROC) analysis showed that area under the curve (AUC) for P-score was 0.75 and AUC for MRI-based T-staging was 0.67.

Figure S4: Predicted biochemical failure in a subgroup of patients who underwent radical prostatectomy (RT) as first- or second- line treatment. Area under the curve (AUC) for P-score and NCCN-score was assessed using receiver operating characteristic (ROC) analysis and the difference between AUC for the two scores was significant at 5 years follow-up (P=0.03).

Figure S5: Relationship between positive biopsy cores (PPBs) (%) and P-score risk stratification (P-score groups 0, 1, and 2) in a combined dataset (n=412) consisting of 96 patients without metastases at diagnosis from the Taiwan cohort and 316 metastasis-free patients from a Swedish cohort (Saemundsson et al 2023).

# Supplementary tables

Table S1

| **NCCN risk group** | **P-score risk group** | **Reclassified patients (number [%])** | **Prostate cancer death** | **Metastasis** |
| --- | --- | --- | --- | --- |
| Favourable intermediate | Low | 1 (20) | - | - |
|  | Intermediate | 3 (60) | - | - |
|  | High | 1 (20) | - | - |
| Unfavourable intermediate | Low | 2 (8) | - | - |
|  | Intermediate | 15 (63) | - | 3 |
|  | High | 7 (29) | - | - |
| High | Intermediate | 5 (8) | - | - |
|  | High | 58 (92) | 8 | 10 |

# Supplementary table legends

Table S1: Reclassification of patients in different NCCN risk groups using the P-score in the subgroup of patients without metastases at diagnosis (n=92).

# References

1. Peng Z, Andersson K, Lindholm J, et al. Operator Dependent Choice of Prostate Cancer Biopsy Has Limited Impact on a Gene Signature Analysis for the Highly Expressed Genes IGFBP3 and F3 in Prostate Cancer Epithelial Cells. Kyprianou N, editor. PLoS ONE. 2014 Oct 8;9(10):e109610.

2. Peng Z, Skoog L, Hellborg H, et al. An expression signature at diagnosis to estimate prostate cancer patients’ overall survival. Prostate Cancer Prostatic Dis. 2014 Mar;17(1):81–90.

3. Prostatype Genomics AB. Prostatype RT qPCR Kit - Instructions for Use. Revision 7. Solna, Sweden. 2021.

4. Söderdahl F, Xu LD, Bring J, Häggman M. A Novel Risk Score (P-score) Based on a Three-Gene Signature, for Estimating the Risk of Prostate Cancer-Specific Mortality. Res Rep Urol. 2022 May;Volume 14:203–17.

5. Röbeck P, Xu L, Ahmed D, et al. P-score in preoperative biopsies accurately predicts P-score in final pathology at radical prostatectomy in patients with localized prostate cancer. The Prostate. 2023 Jun;83(9):831–9.

6. Saemundsson A, Xu LD, Meisgen F, Cao R, Ahlgren G. Validation of the prognostic value of a three-gene signature and clinical parameters-based risk score in prostate cancer patients. The Prostate. 2023 Sep;83(12):1133–40.
